# Comparison of one-stage treatment versus two-stage treatment for the management of patients with common bile duct stones: A meta-analysis

**DOI:** 10.3389/fsurg.2023.1124955

**Published:** 2023-02-03

**Authors:** Shanmao Nie, Shangyu Fu, Kaiyan Fang

**Affiliations:** ^1^ Department of Hepatobiliary Surgery, Luzhou people's Hospital, Luzhou, China; ^2^ Department of Anesthesiology, Luzhou people's Hospital, Luzhou, China

**Keywords:** cholangiolithiasis, endoscopic retrograde cholangiopancreatography, endoscopic sphincterotomy, laparoendoscopic rendezvous, laparoscopic cholecystectomy

## Abstract

**Background:**

Cholelithiasis is a frequently occurring disease in clinic. Due to changes in people's living environments, dietary habits and the aging population, cholelithiasis incidence is increasing. Currently, laparoscopic cholecystectomy (LC) is the preferred treatment for gallbladder stones, but the surgical method for patients with choledocholithiasis is controversial. An endoscopic retrograde cholangiopancreatography (pERCP) is performed preoperatively, followed by LC as the general treatment method. However, pERCP still has some disadvantages, such as prolonged hospital stay, increased incidence of postoperative pancreatitis, and increased duration of anesthesia. Therefore, intraoperative endoscopic retrograde cholangiopancreatography (iERCP) is proposed.

**Objective:**

To compare the efficacy and safety of one-stage treatment and two-stage treatment for the management of patients with cholecystolithiasis and choledocholithiasis.

**Search strategy:**

PubMed, Embase, Web of Science, and Cochrane databases were searched through October 2022. The search terms include cholangiolithiasis/bile duct stones/calculi, endoscopic retrograde cholangiopancreatography/ERCP, endoscopic sphincterotomy/EST, laparoendoscopic rendezvous (LERV), and laparoscopic cholecystectomy/LC.

**Selection criteria:**

For the treatment of patients with cholecystolithiasis and choledocholithiasis in adults, randomized controlled trials (RCTs) comparing LC with iERCP vs. pERCP followed by LC were conducted.

**Data collection and analysis:**

Data extraction and quality assessment were performed by two reviewers. We used Revman version 5.3 to analyze the collected data. The trials were grouped according to the evaluation results such as the overall mortality rate, overall morbidity rate, clearance rate of choledocholithiasis, incidence of pancreatitis, the length of hospitalization, and the length of operation.

**Results:**

9 RCTs (950 participants) were included in this meta-analyses. The overall morbidity rate in LC + iERCP group is lower than that in LC + pERCP group (RR: 0.57, 95% CI = 0.41–0.79, *p *= 0.0008). The clearance rate of choledocholithiasis in LC + iERCP group was almost the same as that in LC + pERCP group (RR: 1.03, 95% CI = 0.98–1.08, *p *= 0.28). The incidence of pancreatitis in LC + iERCP group is lower than that in LC + pERCP group (RR: 0.29, 95% CI = 0.13–0.67, *p *= 0.004). The length of operation of the LC + iERCP group seems to be similar to that of the LC + pERCP group (MD: 16.63 95% CI = −5.98–39.24, *p *= 0.15). LC + iERCP group has a shorter length of hospitalization than that in LC + pERCP group (MD: −2.68 95% CI = −3.39–−1.96, *p *< 0.00001). LC + iERCP group has lower postoperative second ERCP rate than that in LC + pERCP group (RR: 0.13, 95% CI = 0.03–0.57, *p *= 0.006).

**Conclusion:**

Our study suggest that LC + iERCP may be a better option than LC + pERCP in the management of patients with both cholecystolithiasis and choledocholithiasis. This procedure can reduce the overall incidence of postoperative complications, especially the occurrence of postoperative pancreatitis. It could shorten the length of hospital stay, reduce postoperative second ERCP rate.

## Introduction

In the field of hepatobiliary surgery, cholelithiasis is one of the most frequent and frequently occurring diseases. With the change of living environment, people's eating habits and aging, the incidence rate and incidence of diseases increased year by year, which has become a major disease affecting the quality of daily life. Epidemiological reports show that the worldwide incidence rate of cholelithiasis is about 10% to 35%, while gallstone patients account for 75% to 80% of cholelithiasis, of which more than 15% are combined with common bile duct stones (1).

Laparoscopic cholecystectomy is the first choice for the treatment of cholecystolithiasis. For a long time in the past, patients with cholecystolithiasis and choledocholithiasis need open choledochectomy or laparoscopic common bile duct exploration. With the gradual development of endoscopic technology in recent years, some new concepts and surgical methods have been widely recognized.

In 1968, McCunne et al. (2) from Washington University School of Medicine reported ERCP for the first time. Cholangiopancreatography was performed by inserting contrast agent into the duodenal Vater nipple under lateral endoscopic intubation to diagnose biliary and pancreatic diseases. In 1972, British gastroenterologist Cotton (3) first named ERCP based on his own experience and the experience of doctors all over the world, which is still in use today. In 1974, Kawai et al. (4) and Classen et al. (5) successively reported that ERCP was used for duodenal sphincterotomy to remove choledocholithiasis, which opened the era of ERCP treatment.

Laparoscopic cholecystectomy (LC) combined with laparoscopic common bile duct exploration (LCBDE) and endoscopic retrograde cholangiopancreatography (ERCP)/endoscopic sphincterotomy (EST) combined with laparoscopic cholecystectomy (EST + LC) are effective methods for the treatment of cholecystolithiasis combined with choledocholithiasis. The rendezvous technique was described for the first time by Deslandres et al. in 1993., and first completed by Feretis et al. In 1994. During laparoscopic cholecystectomy, a guide wire was inserted from the cystic duct to the duodenal papilla through the common bile duct, so as to guide the tube insertion under duodenoscopy. Finally, the stone basket can be applied through papillary. As a result of rendezvous technology, iERCP has a shorter learning curve and lower technical requirements for laparoscopic surgery, making it less complex to use (6). The aim of this meta-analysis was to evaluate the safety and effectiveness of pERCP vs. iERCP.

## Objective

To compare the efficacy and safety of one-stage treatment and two-stage treatment for the management of patients with cholecystolithiasis and choledocholithiasis.

## Methodology

### Research types

Trials performed and published in English were considered when random assignments were made to one-stage LC combined with iERCP and two-stage pERCP followed by LC.

### Participant types

In our review, only randomized controlled trials in patients with cholecystolithiasis and choledocholithiasis were considered, regardless of clinical symptoms.

### Intervention types

Trials comparing one-stage LC combined with iERCP and two-stage pERCP followed by LC were considered.

### Outcome measure types

Trials were considered if they demonstrated any of the following clinical outcomes:

1. Overall mortality rate (30 days postoperative).
2. Overall morbidity rate (30 days postoperative).
3. Incidence of pancreatitis.
4. Operative time.
5. Postoperative second ERCP rate.
6. Clearance rate of choledocholithiasis.
7. The length of hospital stay (time from admission to postoperative discharge).

### Search strategy

The following keywords were used to search PubMed, Embase, Web of Science, and Cochrane databases for all randomized controlled trials citing pre-operative vs. intra-operative ERCP in patients with cholecystolithiasis and choledocholithiasis: cholangiolithiasis/bile duct stones/calculi, endoscopic retrograde cholangiopancreatography/ERCP, endoscopic sphincterotomy/EST, laparoendoscopic rendezvous (LERV), and laparoscopic cholecystectomy/LC. The reference lists of published and review articles were thoroughly screened manually for duplicate studies and included only those that were relevant. The literature search deadline was October 2022. In PubMed, the detailed literature search strategy is (“cholangiolithiasis”[Title/Abstract] OR “bile duct stones” [Title/Abstract] OR “bile duct calculi” [Title/Abstract]) AND (“endoscopic retrograde cholangiopancreatography” [Title/Abstract] OR “ERCP” [Title/Abstract] OR “endoscopic sphincterotomy” [Title/Abstract] OR “EST” [Title/Abstract]) OR [“laparoendoscopic rendezvous” (Title/Abstract)] AND (“laparoscopic cholecystectomy” [Title/Abstract] OR “LC” [Title/Abstract]). After reviewing the titles and abstracts of relevant literatures retrieved, 430 literatures were obtained initially. Our study included 9 final articles after obtaining the full text and further reading.

### Data collection and analysis

Based on the protocol's criteria, all potential trials were deemed as qualified (7). Literature quality evaluation and data extraction were independently completed by 2 evaluators and cross-checked. First, 2 evaluators independently reviewed all cited titles and abstracts to identify eligible references and eliminate duplications. Secondly, the full text of the qualified literature is read to determine whether it is finally selected. In case of disagreement during cross checking, both parties shall discuss and solve it. Two reviewers independently assessed the quality of the included studies based on the Cochrane Handbook for Systematic Reviews of Interventions, including random sequence generation, concealment of allocation, blinding of personnel and participants, blinding of outcome assessments, incomplete outcome data, and selective reporting. For missing information, we contacted the author by email to supplement it.

### Statistical analysis and publication bias

For dichotomous variables, the risk ratio (RR) and 95% confidence intervals (CI) were calculated. For continuous variables, the therapeutic effect was expressed as mean difference (MD) with 95% CI. If different measurement methods or units were used for the same index and the mean values were significantly different, standardized mean difference (SMD) with 95% CI was used. If there were zero events in both groups, risk difference (RD) was used to avoid excluding zero events. Heterogeneity was assessed using I-square statistics (*I*^2^) (8). An *I*^2^ value greater than 50% indicates significant heterogeneity (8). In case of obvious heterogeneity, sensitivity analysis was performed by excluding each study separately, and then the pooled RR value and its 95% CI were recalculated for the remaining studies to evaluate the stability of the results. The fixed-effects model was used when heterogeneity was low, and the random-effects model was used when heterogeneity was high. *p < 0.05* was considered statistically significant. We used a funnel plot to detect publication bias. Meta-analysis was performed using Review Manager Version 5.3.

## Results

**Figure 1:**
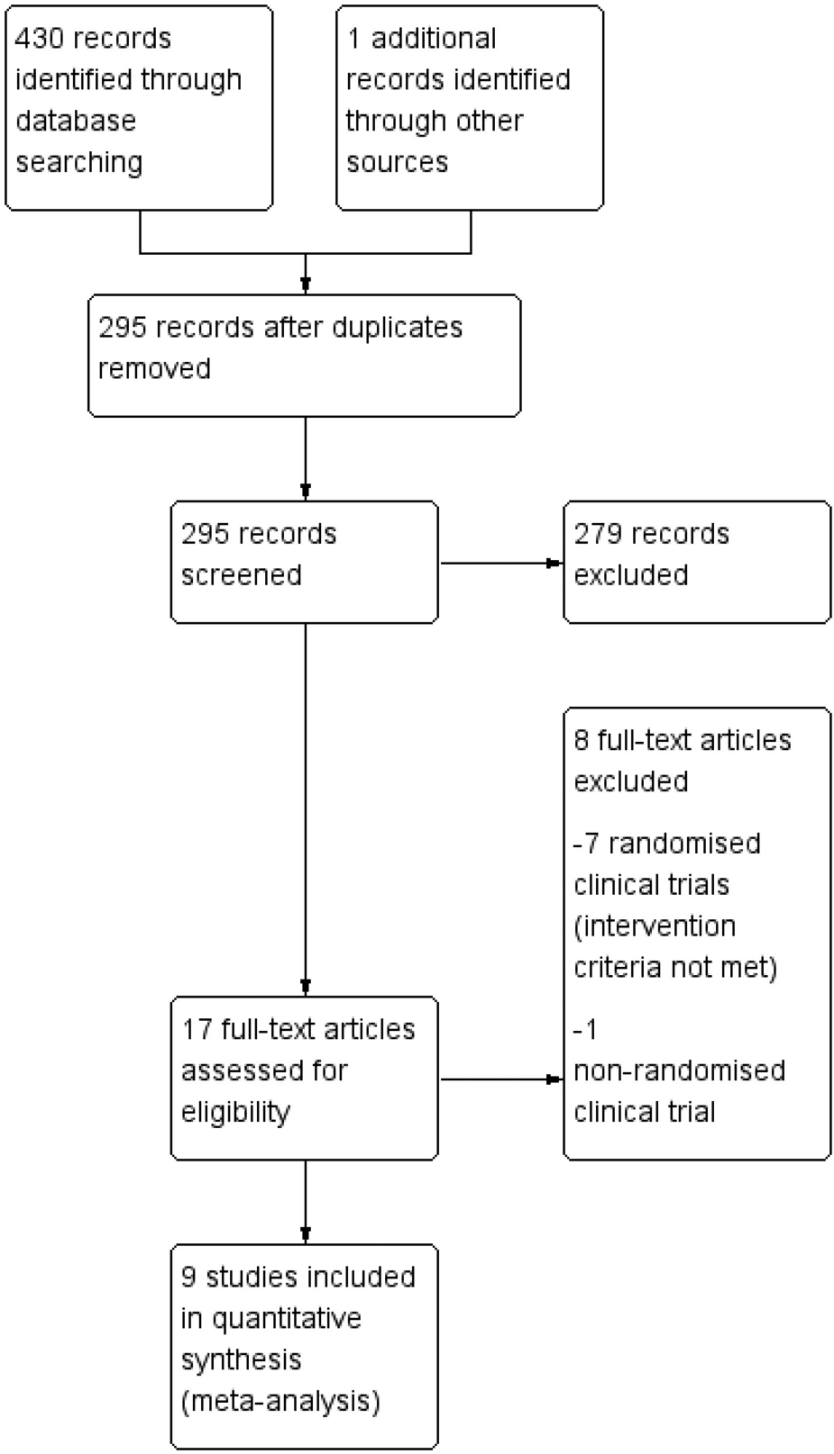
Flow diagram of the search method and selection process.

9 RCTs were included in this meta-analyses. The characteristics of included studies are summarized in Table 1 (9–17). Figure 1 illustrates the process of selecting relevant studies.

**Table 1:** Characteristics of included studies.

Ref.	Approach	*N* (%)	Overall mortality rate *n* (%)	Overall morbidity rate *n* (%)	Success rate of choledocholithiasis clearance *n* (%)	Incidence of pancreatitis *n* (%)	Length of operative time (min)	The length of hospital stay (days)	follow-up time (months)
Lella (11) 2006	LC + iERCP LC + pERCP	60(50) 60 (50)	0 (0) 0 (0)	2 (3.3) 8 (13.3)	58 (96.7) 58 (96.7)	0 (0) 6 (10)	NA	3 ± 0.5 6 ± 1.5	NA
Morino (9) 2006	LC + iERCP LC + pERCP	46 (50.4) 45 (49.5)	0 (0) 0 (0)	3 (6.5) 4 (8.8)	44 (95.7) 36 (80)	1 (2.2) 0 (0)	127 ± 15 80 ± 20.8	4.3 ± 4.25 8 ± 4.75	19 (6–50)
Rabago (12) 2006	LC + iERCP LC + pERCP	59 (48) 64 (52)	0 (0) 0 (0)	5 (8.5) 14 (23)	52 (88.1) 62 (96.9)	1 (1.7) 8 (12.7)	142 ± 58 102 ± 52	5 ± 3 8 ± 5	24
ElGeidie (13) 2011	LC + iERCP LC + pERCP	98 (49.5) 100 (50.5)	0 (0) 0 (0)	4 (4.1) 4 (4)	96 (98) 95 (95)	0 (0) 0 (0)	112 ± 20 90 ± 15	1.3 ± 0.5 3 ± 1.5	NA
Tsovaras (10) 2012	LC + iERCP LC + pERCP	50 (50.5) 49 (49.5)	1 (2) 0 (0)	7 (14) 6 (12.2)	47 (94) 45 (91.8)	0 (0) 0 (0)	95 ± 33.75 79 ± 35	4 ± 4.25 5.5 ± 4.75	NA
Sahoo (14) 2014	LC + iERCP LC + pERCP	42 (50.6) 41 (49.4)	0 (0) 0 (0)	NA	38 (90.5) 29 (71)	0(0) 5(12.2)	NA	6.8 ± 4.25 10.9 ± 4.75	NA
Gonzalez (15) 2016	LC + iERCP LC + pERCP	46 (50.5) 45 (49.5)	0 (0) 0 (0)	0 (0) 6 (13.3)	45 (97.8) 42 (93.3)	0 (0) 1 (2.2)	94.2 ± 63.75 98 ± 52.5	NA	NA
Liu (16) 2017	LC + iERCP LC + pERCP	32 (50.8) 31 (49.2)	0 (0) 0 (0)	17 (53.1) 25 (80.6)	31 (96.9) 30 (96.8)	3 (9.38) 2 (6.45)	129 ± 16.3 151.3 ± 15.2	7.5 ± 1.7 10.6 ± 2.5	NA
Muhammedoglu (17) 2020	LC + iERCP LC + pERCP	39 (47.6) 43 (52.4)	0 (0) 0 (0)	NA	NA	NA	NA	5 ± 3.25 7 ± 4.5	NA

Ref.	Methods	Country	Inclusion criteria	Exclusion criteria
Lella (11) 2006	Parallel randomised controlled clinical trial Randomisation ratio: superiority design	Italy	US and MRI Diagnosis of CBD stone	Age <18 year, pregnancy, previous sphincterotomy, chronic pancreatitis, allergy to propofol and/or fentanyl
Morino (9) 2006	Parallel randomised controlled clinical trial Randomisation ratio: superiority design	Italy	Elevation of serum enzymes + US diagnosis of CBDS or CBD >8–10 mm, no cholangitis and necrotizing pancreatitis	Age <18 year, ASA IV and V, CBD malignancy, previous cholecystectomy, contraindications to MRCP and ERCP, contraindications to laparoscopic surgery
Rabago (12) 2006	Parallel randomised controlled clinical trial Randomisation ratio: superiority design	Spain	US/CT/MRCP diagnosis of CBDS elevated serum enzymes, CBD >8 mm, with cholangitis	Age <18 or >80 year, no contraindication to laparoscopy, no previous upper abdominal surgery, no chronic pancreatitis
ElGeidie (13) 2011	Parallel randomised controlled clinical trial Randomisation ratio: superiority design	Egypt	Clinical assessment + US diagnosis of CBDS or CBD >8 mm + liver chemistry, MRI diagnosis of CBDS, no cholangitis and pancreatitis	Age <18 or >80 year, ASA IV and V, CBD malignancy, pregnancy, previous cholecystectomy, contraindications to MRCP and ERCP, contraindications to laparoscopic surgery, previous upper abdominal surgery, marked liver cirrhosis
Tsovaras (10) 2012	Parallel randomised controlled clinical trial Randomisation ratio: superiority design	Greece	US/MRCP diagnosis of CBDS	Age <18 or >80 year, ASA IV and V, BMI >35, previous upper abdominal surgery, pregnancy
Sahoo (14) 2014	Parallel randomised controlled clinical trial Randomisation ratio: superiority design	India	Diagnosis of gallstone and CBDS	CBDS >12 mm
Gonzalez (15) 2016	Parallel randomised controlled clinical trial Randomisation ratio: superiority design	Cuba	Clinical features + US diagnosis of CBDS or CBD >8 mm + liver function tests, ASA I–III	Age <18, ASA IV and V, previous upper abdominal surgery, previous ERCP, contraindications to ERCP, Contraindications to laparoscopic surgery
Liu (16) 2017	Parallel randomised controlled clinical trial Randomisation ratio: superiority design	China	US/CT/MRCP diagnosis of CBDS, Age ≤75 year, CBDS > 0.2 and <1.5 cm, no upper abdominal surgery, no pancreatitis	Contraindications to ERCP, iodine allergy
Muhammedoglu (17) 2020	Parallel randomised controlled clinical trial Randomisation ratio: superiority design	Turkey	no difficulty in ERCP, US/CT/MRCP diagnosis of CBDS, acute cholecystitis, no malignancy, no history of abdominal surgery, and no contraindications for laparoscopic surgery	Age <16 year, pregnancy, diagnostic ERCP, diffuse peritonitis, perforated gall bladder, malignancy and uncompleted ERCP

NA, not available.

**Figure 2:**
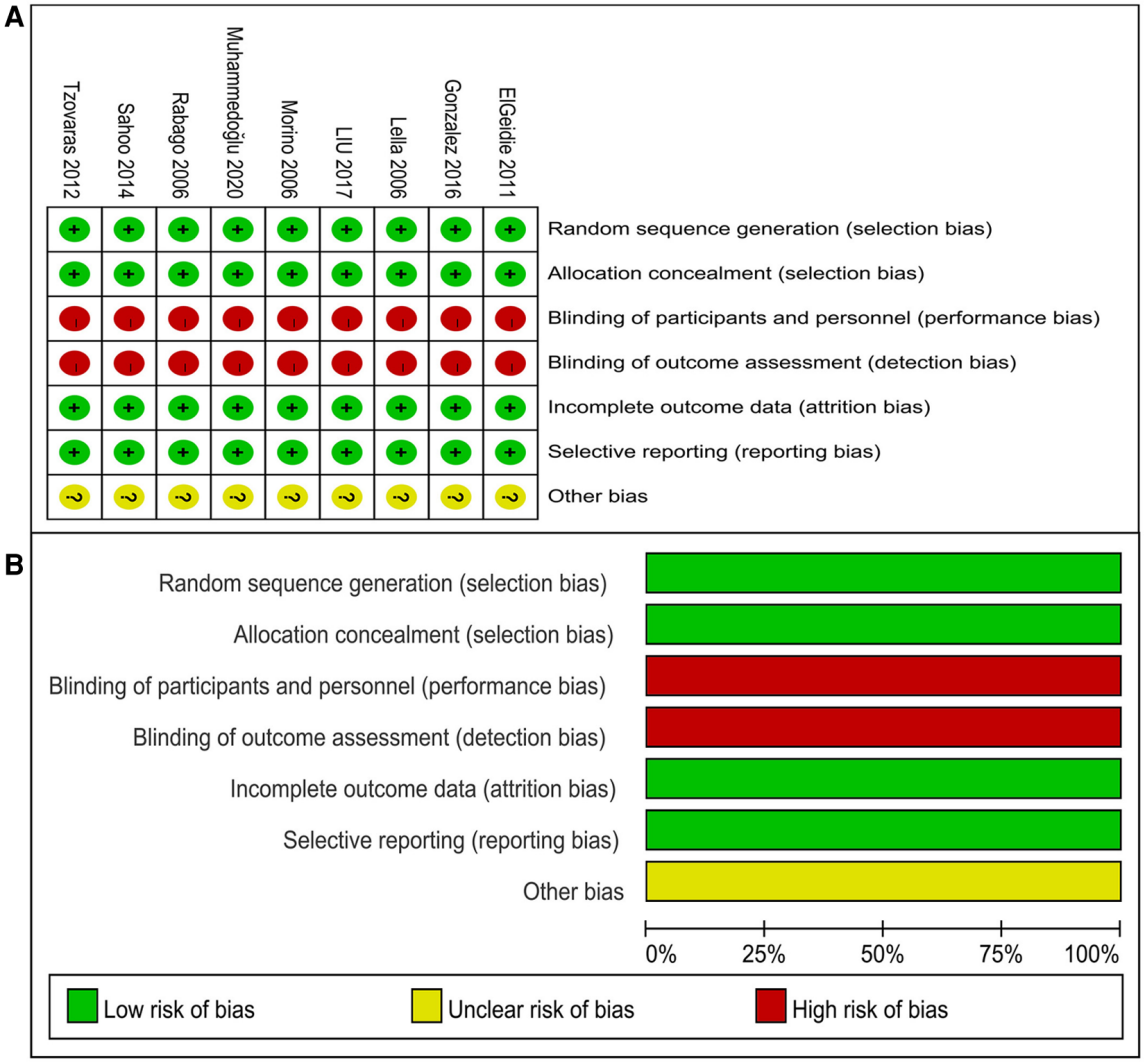
Visualization of bias assessment. (**A**) Summary of risk of bias. (**B**) Risk of bias graph.

We evaluated the quality of the included RCTs according to the Cochrane Collaboration's Tool (see Figures 2A,B).

### Overall mortality rate (30 days postoperative)

All nine trials (950 participants) measured overall mortality. According to the study of Tzovaras et al. (10), the only death in the LERV group was that of a 78-year-old, ASA III patient who was discharged on the 2nd postoperative day after having a successful rendezvous procedure. As a result of an intra-abdominal abscess that required drainage, the patient was re-hospitalized on day seven. On day 18, the patient died of multiple organ failure.

### Overall morbidity rate (30 days postoperative)

**Figure 3:**
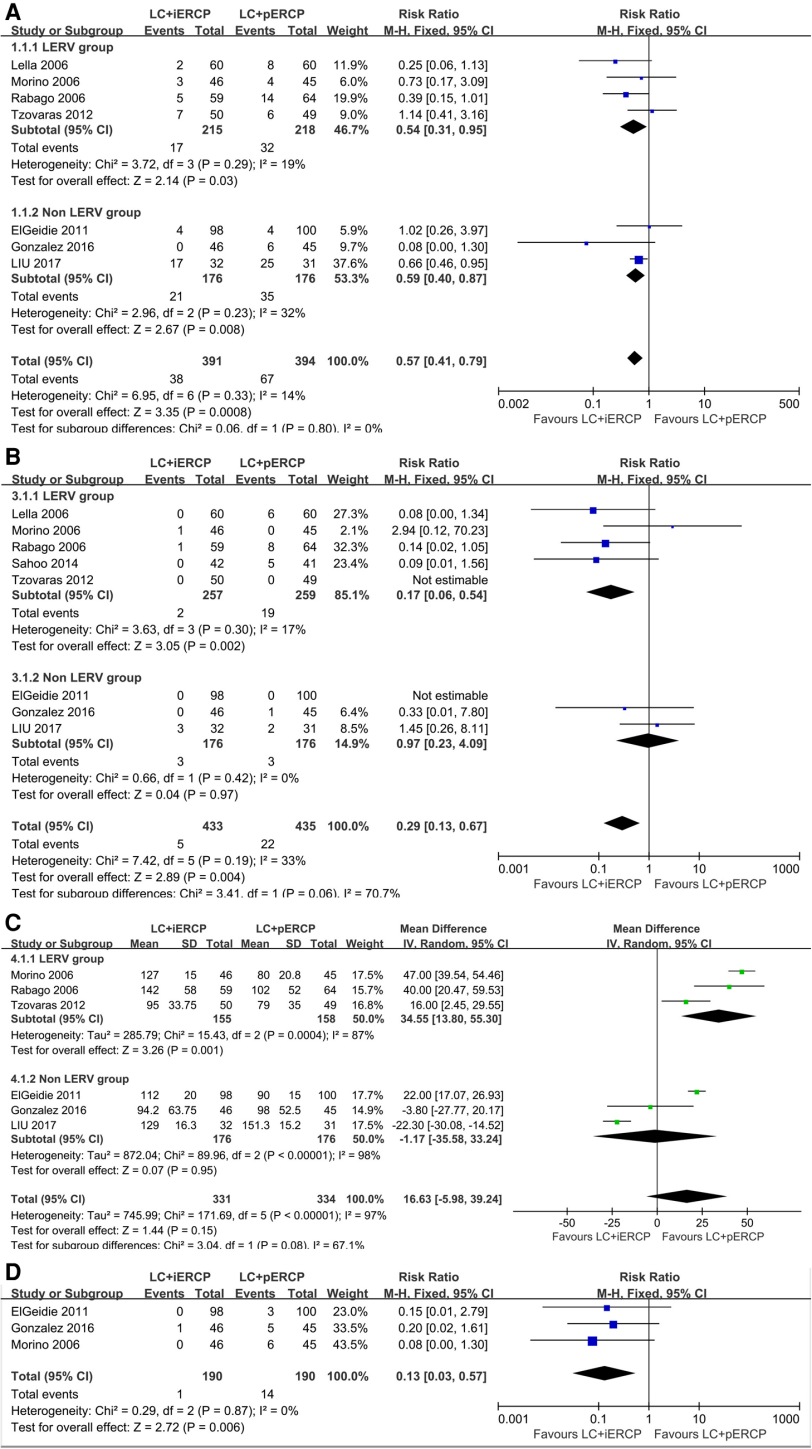
Forest plot of clinical outcomes. (**A**) Overall morbidity rate. (**B**) Incidence of pancreatitis. (**C**) Operative time. (**D**) Postoperative second ERCP rate.

Seven trials (785 participants) reported lower overall morbidity in the LC + iERCP group than that in the LC + pERCP group (RR: 0.57, 95% CI = 0.41–0.79, *p *= 0.0008) (Figure 3A). Statistical analysis showed that overall mortality were statistically significant between two groups, favoring LC + iERCP group. There was no heterogeneity among trials (*I*^2 ^= 14%). The funnel plot has basically symmetrical sides, indicating no apparent publication bias.

*Conclusion* The overall morbidity rate in LC + iERCP group is lower than that in LC + pERCP group.

### Incidence of pancreatitis

Eight trials (868 participants) reported lower incidence of pancreatitis in the LC + iERCP group than that in the LC + pERCP group (RR: 0.29, 95% CI = 0.13–0.67, *p *= 0.004) (Figure 3B). Statistical analysis showed that incidence of pancreatitis were statistically significant between two groups, favoring LC + iERCP group. In subgroup analysis, we found that in the LERV group, the incidence of pancreatitis in one-stage group was lower than that in two-stage group (RR: 0.17, 95% CI = 0.06–0.54, *p *= 0.002), while in the non LERV group, the incidence of pancreatitis in LC + iERCP group was similar to that in LC + pERCP group (RR: 0.97, 95% CI = 0.23–4.09, *p *= 0.97). The heterogeneity among trials was low (*I*^2 ^= 33%). The funnel plot has basically symmetrical sides, indicating no apparent publication bias.

*Conclusion* The incidence of pancreatitis in LC + iERCP group is lower than that in LC + pERCP group.

### Operative time

Six trials (665 participants) reported similar operative time between the two groups (MD: 16.63 95% CI = −5.98–39.24, *p *= 0.15) (Figure 3C). Between the two groups, there was no significant difference. In subgroup analysis, we found that in the LERV group, the operative time in one-stage group was shorter than that in two-stage group (MD: 34.55 95% CI = 13.80–55.30, *p *= 0.001), while in the non LERV group, the operative time in LC + iERCP group was similar to that in LC + pERCP group (MD: −1.17 95% CI = −35.58–33.24, *p *= 0.95). The heterogeneity among the trials is high (*I^2^* = 97%). We found that heterogeneity was still high after excluding studies one by one. We did not find the source of heterogeneity after intensive reading of the six studys. The funnel plot has basically symmetrical sides, indicating no apparent publication bias.

*Conclusion* The length of operation of the LC + iERCP group seems to be similar to that of the LC + pERCP group.

### Postoperative second ERCP rate

Only three trials (380 participants) reported lower postoperative second ERCP rate in the single-stage group compared to the two-stage group (RR: 0.13, 95% CI = 0.03–0.57, *p *= 0.006) (Figure 3D). There was a statistically significant difference between the two groups, favoring LC + iERCP. The heterogeneity among trials was low (*I*^2 ^= 0%).

*Conclusion* LC + iERCP group has lower postoperative second ERCP rate than that in LC + pERCP group.

### Clearance rate of choledocholithiasis

**Figure 4:**
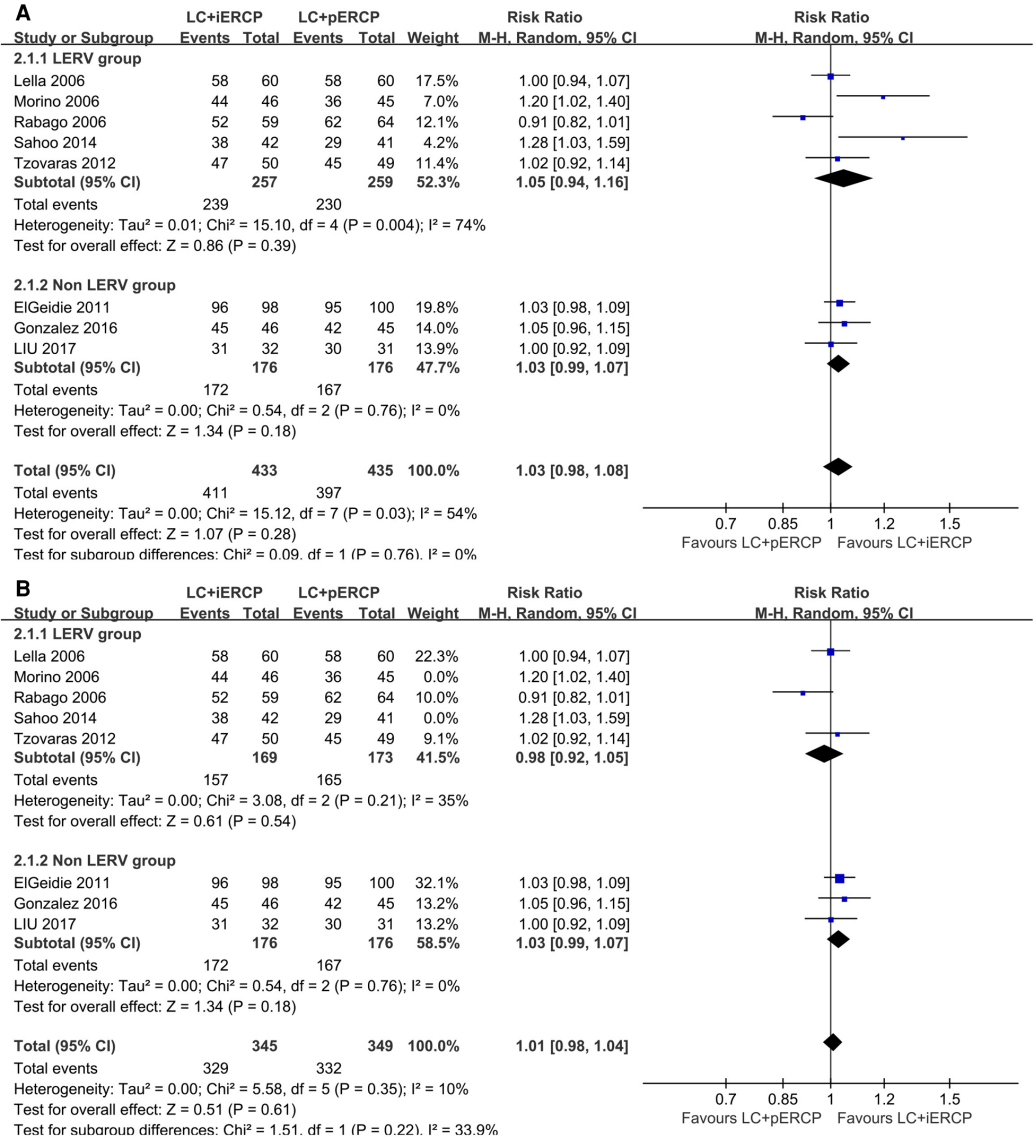
Forest plot of clearance rate of choledocholithiasis. (**A**) the pooled RR value of eight trials. (**B**) the pooled RR value of six trials after excluding two studys (Morino 2006 and Sahoo 2014).

Eight trials (868 participants) reported similar clearance rate of choledocholithiasis between the two groups (RR: 1.03, 95% CI = 0.98–1.08, *p *= 0.28) (Figure 4A). There was no significant difference between the two groups. There is significant heterogeneity among the trials (*I^2^* = 54%). We found that heterogeneity was significantly reduced by excluding two studys (Morino 2006 and Sahoo 2014) (RR: 1.01, 95% CI = 0.98–1.04, *p *= 0.61, *I^2^* = 10%) (Figure 4B). We considered that inclusion and exclusion criteria may be the main cause of heterogeneity after intensive reading of the two studys. The funnel plot has basically symmetrical sides, indicating no apparent publication bias.

*Conclusion* The clearance rate of choledocholithiasis in LC + iERCP group was almost the same as that in LC + pERCP group.

### The length of hospital stay

**Figure 5:**
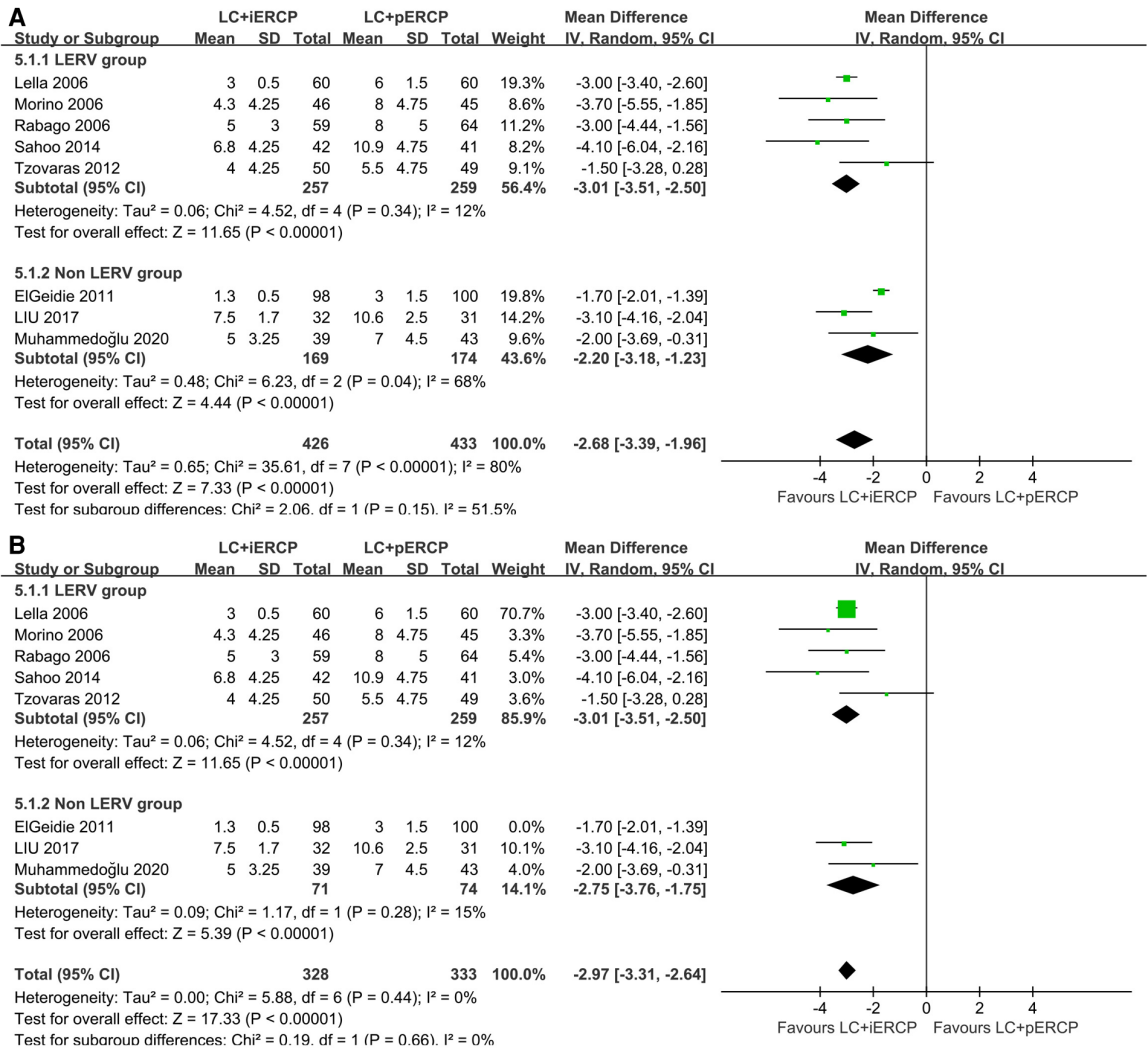
Forest plot of the length of hospital stay. (**A**) the pooled MD of eight trials. (**B**) the pooled MD of seven trials after excluding one study (ElGeidie 2011).

Eight trials (859 participants) reported a shorter length of hospital stay in the LC + iERCP group compared to the LC + pERCP group (MD: −2.68 95% CI = −3.39–−1.96, *p *< 0.00001) (Figure 5A). There was a statistically significant difference between the two groups, favoring LC + iERCP. There is significant heterogeneity among the trials (*I^2^* = 80%). Heterogeneity was significantly reduced after the exclusion of one study (ElGeidie 2011) (MD: −2.97 95% CI = −3.31–−2.64, *p *< 0.00001, *I^2^* = 0%) (Figure 5B). We considered that inclusion and exclusion criteria may be the main cause of heterogeneity after intensive reading of the study. The funnel plot has basically symmetrical sides, indicating no apparent publication bias.

*Conclusion* LC + iERCP group has a shorter length of hospital stay than that in LC + pERCP group.

## Discussion

Cholelithiasis is one of the frequent biliary diseases both at home and abroad, including gallbladder stones and choledocholithiasis. A gallstone passage from the cystic duct into the common bile duct is the most common cause of choledocholithiasis. Nearly 18% of patients hospitalized for cholecystolithiasis have choledocholithiasis (18), and within the first year following cholecystectomy, approximately 3.8% will experience choledocholithiasis symptomsy (19). The traditional surgical methods are open cholecystectomy and choledocholithotomy. These treatment methods are effective. Postoperative indwelling tube can reduce the incidence of bile leakage. Reserving stone removal channel is an effective method for the treatment of postoperative residual bile duct stones. However, its disadvantages are large trauma, slow recovery, intolerance to high-risk patients, long hospital stay, and catheter-related complications (20). With the progress of medical technology, people are more and more inclined to ensure the treatment effect. At the same time, the surgical method of small trauma and fast healing is adopted. Therefore, minimally invasive diagnosis and treatment came into being and has been widely used (21).

At present, LC + LCBDE and ERCP + LC are the two main minimally invasive surgical methods for the treatment of cholecystolithiasis and choledocholithiasis (22). Laparoscopic common bile duct exploration requires special expertise, which is not usually widely available, leaving ERCP as the most popular treatment for common bile duct stones. At present, there are three surgical methods for LC + ERCP in the treatment of cholecystolithiasis combined with choledocholithiasis: preoperative, intraoperative and postoperative ERCP. However, which method is better is still controversial. At present, preoperative ERCP is the most common clinical approach. However, this traditional two-stage procedure have some disadvantages. For example, before performing a cholecystectomy, patients have to wait for one to two days, during which time gallstones may enter the CBD and patients may suffer more pain and stay in the hospital longer. However, one-stage approach may have some advantages in these aspects.

In recent years, more and more studies have focused on the feasibility of one-stage procedure. The rapid development of medical technology and the accumulation of surgical and endoscopic experience make this method applied in clinic. Mattila et al. (23) compared the advantages of one stage LC + LCBDE to the 2 stage pre-ERCP + LC. The study found that the one stage LC + LCBDE had a higher CBD clearance rate, fewer procedures, shorter hospital stays, and lower hospital costs. The retrospective study concluded that one-stage LC + LCBDE might be an attractive treatment strategy for cholecystocholedocholithiasis if local expertise and resources are available. In patients with cholecystolithiasis and choledocholithiasis, Saito et al. (24) demonstrated the safety and feasibility of one-stage cholecystectomy after ERCP. These studys highlight the advantage of one-stage procedure.

Vakayil et al. (25), in a retrospective study, compared the benefits of the LC + LCBDE procedure with the LC + iERCP procedure for the management of choledocholithiasis. The study showed a statistically significant difference between the two procedures as for the mean operative time (*p *< 0.001). The study showed low morbidity and mortality in both groups. The study concluded that centers with extensive endoscopic experience might be more inclined to LC + iERCP because of its ease of access and shorter operative time.

There is still disagreement about the order of ERCP and LC for execution, as well as the follow time between the procedures. Several articles agree that LC should be carried out shortly after ERCP. It was reported that early LC was safe within 72 h after ERCP (26). However, some scholars believed that the time interval might lead to the emergence of new secondary common bile duct stone (27). Other disadvantages include longer hospital stay, increased treatment costs and readmission rate (28–31).

Our paper aimed at comparing the efficacy and safety of the single-stage and two-stage procedure. The main indicator of the therapeutic effect of common bile duct stones is whether the stones are removed. Chester Tan et al. (32) reported the CBD stones clearance rate in the one-stage group and two-stage group was 93.3% and 89.4%, respectively, which had a similar result to previous research results. In our study, we also found the clearance rate of choledocholithiasis in LC + iERCP group was almost the same as that in LC + pERCP group. We analyzed that the main reason was due to the continuous improvement of endoscopic technology and equipment. Only 1 patient death was reported in the 9 studies we included, who died on postoperative day 18 due to multiple organ failure. The patient's death is not directly related to the operation itself. The main reasons for the death of this patient were related to the patient's age, ASA status and delayed readmission (the patient was readmitted after 48 h of fever at home).

The main complications associated with ERCP include bleeding, perforation, cholangitis, cholecystitis, gastric ulcer, hyperamylasemia and pancreatitis. The main complications associated with LC include biliary tract injury, bile leak, bleeding, abdominal fluid, pneumonia, etc. Our study found lower overall morbidity rate in LC + iERCP group than that in LC + pERCP group (9.72% *vs.* 17.01%). We analyzed that the main reasons may be due to different anesthesia methods and the length of operation. Pancreatitis is one of the most common and serious postoperative complications of ERCP, with an incidence of 1%–5% (33). An incidence of pancreatitis of 3.11 percent was observed in our study. The risk factors inducing PEP are not yet clear. At present, most current studies have suggested two main aspects: one is patient-related factors, including female patients, age less than 50 years, pancreatic division, Oddi sphincter dysfunction, recurrent pancreatitis (at least 2 times) and history of PEP; the other is surgery-related factors, consisting of a precut sphincterotomy or sphincterotomy, excessive papillary injury, difficulty in catheterization or multiple catheterization attempts, failure of common bile duct lithotomy, history of pancreatic duct injection or endoscopic operation, deep placement of guide wire into pancreatic duct and no pancreatic duct stent placement (34–36). The 2014 European Society for Digestive Endoscopy (ESGE) Guidelines for PEP prevention pointed out that Oddi sphincter dysfunction, female patients, history of pancreatitis, catheterization attempt >10 min, pancreatic duct injection history, and guide wire length >1 cm into pancreatic duct were clear PEP risk factors, and the rest were possible risk factors (37). PEP can prolong the hospital stay and increase the medical cost of ERCP patients. At the same time, the risk of other complications and even death is significantly increased. In our study, we found the incidence of pancreatitis in LC + iERCP group is lower than that in LC + pERCP group. In subgroup analysis, we found that in the LERV group, the incidence of pancreatitis in LC + iERCP group was lower than that in LC + pERCP group, while in the non LERV group, the incidence of pancreatitis in LC + iERCP group was similar to that in LC + pERCP group. The main reason for our analysis was that the LERV group reduced the number of catheterizations.

A retrospectively cohort study (38) found that the one-stage group experienced a longer total operative time compared to the two-stage group (139.8 ± 46.8 min vs. 107.7 ± 40.6 min, *p* < 0.05). The retrospective study analysed this difference may because of the difficulty of LERV technology. In our study, the operative time, as assessed in six of the nine RCTs, was about the same between the two groups. However, in the subgroup analysis, we found that the LERV group had shorter operative time. The main reason for our analysis was that the application of LERV technology significantly shortened the intubation time of ERCP. Eight of the nine trials reported a shorter length of hospital stay in the LC + iERCP group compared to the LC + pERCP group. In the two-stage group, the interval time between the two procedures was generally 1 to 3 days, which led to a longer stay for the patient and a lack of compliance on their part. And the higher overall morbidity rate in two-stage group may also lead to longer hospital stays. Our results suggested that one-stage group had lower postoperative second ERCP rate than that in two-stage group. The most important reason was that gallbladder stones fell into the common bile duct during the interval time between operations.

Although single-stage surgery has a wide application prospect, we also need to pay attention to some problems in the operation process. First, endoscopic gas injection should be minimized during ERCP operation, and the residual gas in the intestine and stomach should be sucked as far as possible at the end of the operation. Second, we should try our best to complete the ERCP operation in the position of the LC, and minimize the movement of the patient's position to avoid some potential risks. Third, for some special cases such as Mirizzi syndrome, LERV technology may not be suitable, in which case we need traditional catheterization combined with Spyglass to complete the operation. Fourth, for the removal of large common bile duct stones, lithotripsy followed by balloon dilation may be a better choice.

According to our study, LERV technology can significantly reduce operation time and the occurrence of postoperative pancreatitis; therefore, we should incorporate this technology into one-stage surgeries as much as possible. Moreover, we recommend that standardized, effective procedures be developed to ensure the operation's success.

Of course, our research has some shortcomings: First, the inclusion criteria and exclusion criteria of the included literatures were not consistent; Second, most of the literature did not provide follow-up time and long-term stone recurrence rate; Third, the heterogeneity of the included literatures was large and no causes were found; Fourth, most of the included literature was too old, and there was little literature on the comparison between the two groups in the last 5 years; Sixth, The number of documents included was small; Seventh, none of the included literatures used a complication scale.

## Conclusion

Our study suggest that LC + iERCP may be a better option than LC + pERCP in the management of patients with both cholecystolithiasis and choledocholithiasis. This procedure can reduce the overall incidence of postoperative complications, especially the occurrence of postoperative pancreatitis. It could shorten the length of hospital stay, reduce postoperative second ERCP rate.
