# Supplementary figures and images for: Comparison of one-stage treatment versus two-stage treatment for the management of patients with common bile duct stones: A meta-analysis
Source: Front Surg. 2023 Feb 3;10:1124955. doi: 10.3389/fsurg.2023.1124955 (PMC9935819; doi:10.3389/fsurg.2023.1124955)

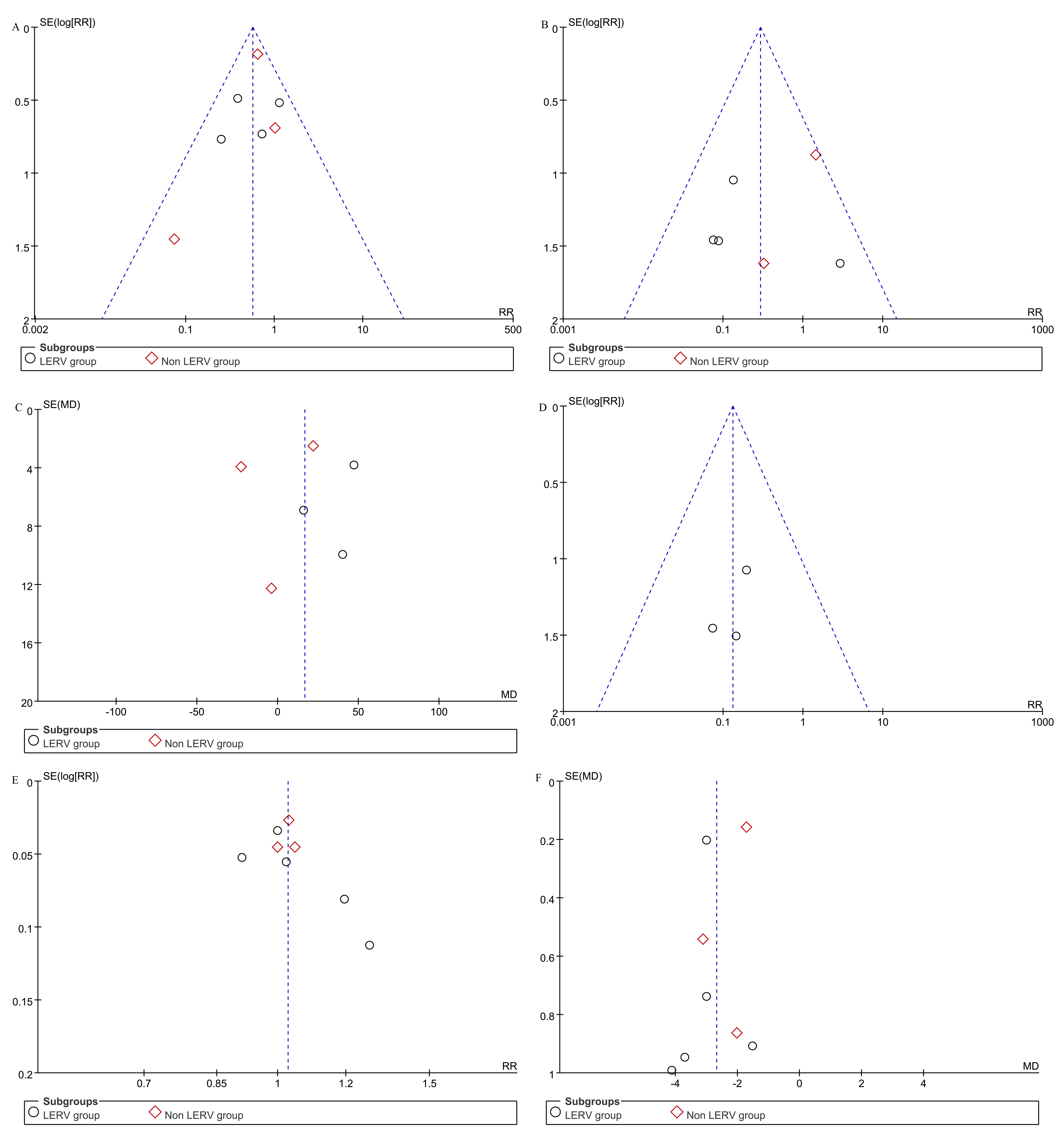

Supplement: Supplementary file 1 [file Image1.jpeg]
